# Recent Advances in Field Effect Transistor Biosensors: Designing Strategies and Applications for Sensitive Assay

**DOI:** 10.3390/bios13040426

**Published:** 2023-03-27

**Authors:** Ruisha Hao, Lei Liu, Jiangyan Yuan, Lingli Wu, Shengbin Lei

**Affiliations:** 1 Tianjin Key Laboratory of Molecular Optoelectronic Science, Department of Chemistry, School of Science, Tianjin University, Tianjin 300072, China; 2 Medical College, Northwest Minzu University, Lanzhou 730000, China

**Keywords:** field effect transistor, biosensors, microfluidics, multiplexing, integration

## Abstract

In comparison with traditional clinical diagnosis methods, field–effect transistor (FET)–based biosensors have the advantages of fast response, easy miniaturization and integration for high–throughput screening, which demonstrates their great technical potential in the biomarker detection platform. This mini review mainly summarizes recent advances in FET biosensors. Firstly, the review gives an overview of the design strategies of biosensors for sensitive assay, including the structures of devices, functionalization methods and semiconductor materials used. Having established this background, the review then focuses on the following aspects: immunoassay based on a single biosensor for disease diagnosis; the efficient integration of FET biosensors into a large–area array, where multiplexing provides valuable insights for high–throughput testing options; and the integration of FET biosensors into microfluidics, which contributes to the rapid development of lab–on–chip (LOC) sensing platforms and the integration of biosensors with other types of sensors for multifunctional applications. Finally, we summarize the long–term prospects for the commercialization of FET sensing systems.

## 1. Introduction

In the case of highly contagious and hidden viruses which spread recklessly around the world at an alarming rate, detecting and controlling an epidemic as early as possible can effectively reduce the harm caused to society by public health events to a large extent [1]. Therefore, early non–invasive diagnosis and the immediate detection of biomarkers has become a research hotspot. How to realize simple, rapid, sensitive and low–cost detection of biological target analytes such as viruses and various proteins has also become a major problem in the field of biosensors. 

A biosensor is a device that is sensitive to biological substances and which can convert concentration signals into readable signals of light, electricity, and magnetism. It generally consists of biologically sensitive probes performing the identification of elements (enzymes, antibodies, antigens, nucleic acids and other biologically active substances), appropriate physical and chemical transducers (oxygen electrodes, field effect transistors etc.), and an analysis system composed of a signal amplification device [2]. Common biosensors include optical biosensors, thermal biosensors, resistive biosensors, and semiconductor biosensors. Particularly, FET biosensors have shown great technical potential in biomarker detection platforms due to their simple operation, high sensitivity, fast response speed, real–time signal amplification, easy miniaturization, and integration for high–throughput screening, which has caused them to become a promising candidate for various biosensing applications [3,4,5,6]. The main principle of FET biosensors for biological detection is that the bio–sensitive probe should specifically bind with the target analyte and generate charged ions, which will further induce the change of carriers in the channel material [7]. With the change of various electrical output parameters, such as mobility (μ), threshold voltage (V_th_), on/off ratio (I_on_/I_off_) and source–drain currents (I_ds_), the signals can also be effectively transmitted into electrical signals and amplified even in complex biological systems, thereby realizing the quantitative detection of biological substances [8]. Many FET biosensors have been successfully used for the sensitive detection of proteins, glucose, DNA, and cells, illustrating the rapid development of this exciting research field [9].

For FET biosensors, the realization of high efficiency signal transduction not only depends on optimizing the geometry of devices and the functionalization methods of devices, but also heavily relies on the development of semiconductor materials. Furthermore, detection for single analytes alone is far from sufficient to reach the required accuracy for early disease detection. Consequently, biosensor multiplexing has been developed to detect one analyte in multiple parallel channels or to detect multiple analytes simultaneously to improve accuracy and repeatability, and this multiplexing has been the key to the application of advanced FET biosensors in the practical medical field. FETs are small in size and compatible with traditional semiconductor microfabrication processes, so they could be integrated into microfluidic platforms. Integrating the microfluidics and immunoassays into lab–on–chip (LOC) devices can help detect biomarkers in a shorter analyzing time, with less reagent volume and lower power consumption automatically, which can contribute to developing handheld, miniaturized, medical diagnostic testing platforms. Therefore, we will discuss recent progress regarding the aspects mentioned above in this mini review and summarize the long–term prospects for the commercialization of FET sensing systems.

## 2. Biosensor Designing

### 2.1. Device Structures

**Figure 1:**
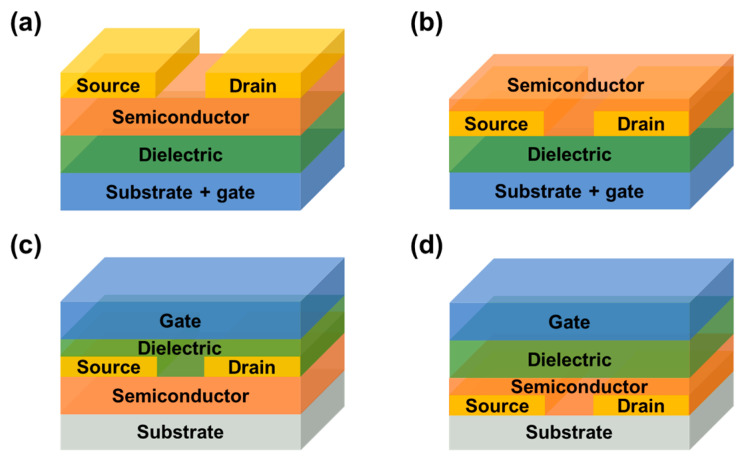
Schematic representation of four configurations of FETs. (**a**) Bottom–gate top contact (BGTC). (**b**) Bottom–gate bottom contact (BGBC). (**c**) Top–gate top contact (TGTC). (**d**) Top–gate bottom contact (TGBC).

FETs are mainly composed of three electrodes (gate, source and drain), an insulating layer and a semiconductor layer [10]. The device is “energized” only when the gate voltage reaches the “threshold voltage” (V_th_). When it is above V_th_, carriers flow along the channel between the source and drain. Therefore, the device state of “on” or “off” is related to the relative magnitude of the gate bias voltage (V_g_) applied to the FET and V_th_. According to the relative position of the electrode and the semiconductor layer, there are four basic structures of FET, as shown in Figure 1. 

In these device structures, when a metal and semiconductor are in contact, due to the difference in work function, free electrons will transfer from the metal to the semiconductor, or vice versa, forming a space charge region. Then, energy band edges in the semiconductor are shifted continuously because of an electric field generated by the charge transfer, which is called metal/semiconductor–contact–induced band bending. When an extra electric field is applied to the metal, an electric field is built between the metal and the semiconductor, and because of insufficient shielding by the charge carriers of low concentration, the electric field is penetrated into the near surface region of the semiconductor, causing field–effect–induced band bending [11]. In addition, charge transport within a device is also strongly influenced when charged molecules are adsorbed on a semiconductor surface. Specifically, when a molecule approaches the semiconductor surface, the potential energy gradient of electrons and holes in the near–surface region of the semiconductor is modified by adsorbed molecules, forming Helmholtz layers on the semiconductor surface and causing conduction and valence bands to bend. Therefore, due to the band bending effect, the efficiency of charge transfer from the semiconductor to the adsorbed molecule will be affected [12].

**Figure 2:**
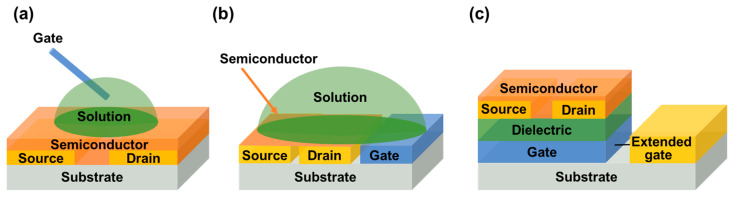
Schematic representation of three configurations of EGFETs. (**a**) Top–gate structure. (**b**) Side–gate architecture. (**c**) Extended–gate structure.

In recent years, in order to expand the application of FETs, researchers have replaced the traditional insulating layer materials with electrolytes, such as polymers or ionic liquids, and allowed contact with the gate electrodes to fabricate electrolyte–gated transistors (EGTs). Considering that electrochemical switching and field–effect modulation in semiconductor channels may often coexist, we will only discuss electrolyte–gate field–effect transistors (EGFETs) operating fully in field–effect mode here. Different from traditional FETs, the channel current of EGFETs is regulated by the gate electrode through the electrolyte solution, so that EGFETs show higher gate capacitance and lower operation voltage (less than 1V). In the EGFETs, depending on the position of the gate electrode relative to the semiconductor channel, there are several common geometric structures, as shown in Figure 2.

In the first structure (Figure 2a), the manually placed probe gate electrode is located above the semiconductor channel. For example, Horowitz’s group used Au as the gate electrode and a simple water droplet as an insulating layer for the first time and fabricated a water–gate organic field–effect transistor (WGOFET) [13]. As water is the natural environment for livings, it is extremely suitable for detecting biological molecules. Following this, Kergoat et al. used WGOFETs for DNA testing. According to the formula calculation, the Debye length in PBS was 0.76 nm and a significant amount of negative charge of DNA was located outside of the Debye length, but it could be increased to 206 nm in deionized water at room temperature, which solved the problem of shielding DNA negative charge in high ion concentration solutions [14].

Because the position of a manually placed probe is arbitrary in the structure of WGOFETs, it is not easy to integrate such probe gate electrode structures into microfluidic channels. Consequently, side–gate architecture (Figure 2b) was proposed, in which the gate is on the same plane as the semiconductor channel. The main advantages of this structure were that the gate electrode position was highly controllable, the fabrication of devices was simplified greatly, and the source, drain, and gate electrodes could be simultaneously deposited by using a single pattern process. Kim et al. used liquid coplanar gate graphene FETs to detect and distinguish single strand (SS) and double strand (DS) DNA molecules [15].Compared with the traditional bottom–gate graphene field–effect transistors (GFETs), liquid coplanar–gate graphene FETs showed higher DNA detection sensitivity [16].

Considering that most of the research on biosensors is based on “bottom gate” or “solution gate” and the sensing region is placed on the semiconductor which is sensitive to factors such as water and oxygen, some researchers proposed the “extended gate” structure (Figure 2c) so as to protect semiconductors, which separated the sensing area from the transistor itself. Minamiki et al. achieved the label–free detection of phosphoproteins (α–casein) using Zn^II^–DPA functionalized extended–gate electrodes; the detection of phosphoproteins can be applied in the fields of medicine and bioanalytical chemistry [17]. Zhang also reported an extended–gate organic FET sensing platform for exploiting the difference in weak steric interaction between cationic phenylcarbamoylated–CD and essential amino acids, which can be amplified strongly via organic field–effect transistors (OFETs), and it exhibited good chiral resolution for six essential amino acids [18]. This study provided a new direction for the molecular chirality study of natural amino acids.

### 2.2. Device Functionalization Methods

In addition to the rational design of the structure of devices, adopting suitable device functionalization methods was also important to achieve high sensitivity and selective detection of biological target analytes. The functionalization methods can be divided into two categories: physical functionalization methods and chemical functionalization methods [19].

#### 2.2.1. Physical Functionalization Methods

**Figure 3:**
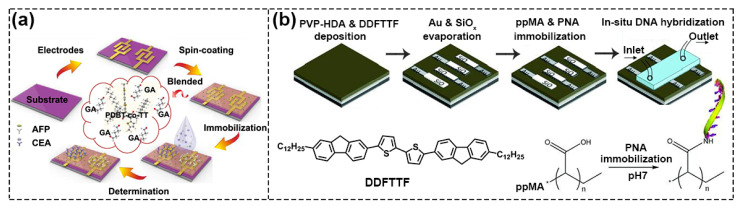
Schematic diagram of physical functionalization methods. (**a**) Flow chart of PDBT–co–TT/GA blend films. Reproduced with permission from [21]. Copyright 2021, American Chemical Society. (**b**) Process of deposition of a functional layer containing carboxyl groups on DDFTTF semiconductor surface by polymerization of MA monomer. Reproduced with permission from [22]. Copyright 2010, John Wiley and Sons.

The physical functionalization of semiconductors is to connect semiconductors and biological acceptors (which refers to any chemicals that have a recognition unit or reaction site with the target analyte) only through simple weak interaction such as van der Waals force and electrostatics interaction etc., instead of covalent bonds. One strategy is to blend them directly [20]. As shown in Figure 3a, Sun et al. chose glutaraldehyde (GA) as the dopant and achieved lower V_th_ and higher μ when adding 10% GA crosslinker to poly{2,2′–[(2,5–bis(2–octyldodecyl)–3,6–dioxo–2,3,5,6–tetrahydropyrrolo[3,4–c] pyr–role–1,4–diyl)] dithiophene–5,5′–diyl–alt–thieno[3,2–b] thiophen–2,5–diyl} (PDBT–Co–TT) solution. The main reasons for the obvious improvement in device performance were: (i) the gelation behavior of PDBT–co–TT polymer was effectively suppressed by the GA crosslinker, thus forming a better charge transport film; (ii) GA cross–linking agent acted as dopant and its strongly polar–CHO group facilitated the accumulation and transportation of charges, which contributed to improving the performance [21].

**Figure 4:**
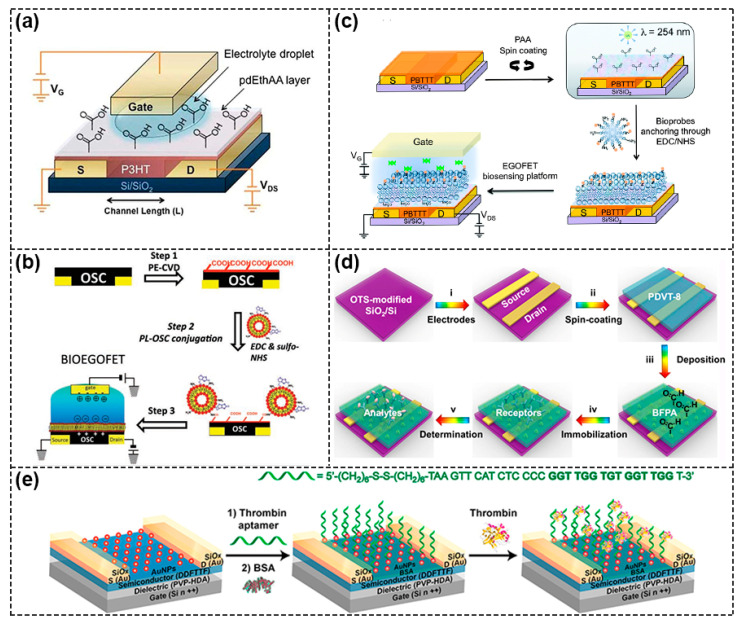
Schematic diagram of physical functionalization methods. (**a**) Schematic of a functional layer containing carboxyl groups deposited on the surface of P3HT semiconductor. Reproduced with permission from [24]. Copyright 2013, WILEY–VCH Verlag GmbH & Co. KGaA, Weinheim. (**b**) Procedure of the PECVD method to introduce carboxyl functional layer onto the OSC surface and immobilization of phospholipid (PL) molecule for biological modification. Reproduced with permission from [25]. Copyright 2013, John Wiley and Sons. (**c**) Schematic diagram of introducing the carboxyl functional layer onto PBTTT surface, including the spin coating of PAA layer on PBTTT surface and subsequent UV–assisted cross–linking process. Reproduced with permission from [26]. Copyright 2015, Royal Society of Chemistry (**d**) Schematic diagram of BFPA layer prepared by spin–coating method as both protective layer and functional layer. Reproduced with permission from [27]. Copyright 2021, American Chemical Society. (**e**) Schematic of using deposited gold nanoparticles (AuNPs) on DDFTTF semiconductor surfaces as functional layers to provide binding sites for DNA aptamer. Reproduced with permission from [30]. Copyright 2013, American Chemical Society.

Because blending often adversely affects the performance of FETs, researchers have tried to directly deposit the acceptor on the semiconductor to form a bilayer structure to physically functionalize the semiconductor, and the most commonly used method is Plasma Enhanced Chemical Vapor Deposition (PECVD). For example, Bao’s group used this method to deposit maleic anhydride (MA) monomer in a plasma chamber onto the surface of 5,5′ –bis–(7–dodecyl–9H–fluoren–2–yl)–2,2′ –bithiophene (DDFTTF) semiconductor for DNA detection. MA was polymerized on the surface to form a 5 nm–thick ultrathin film containing carboxyl groups to allow for the covalent attachment of the peptide nucleic acid (PNA) strands (Figure 3b) [22,23]. As displayed in Figure 4a, Torsi’s research group used ethylene and acrylic acid vapor as a precursor and used glow discharge in a plasma reactor to induce polymerization on the surface of P3HT [24]. Because the formed carboxyl functional layer was a hydrophilic layer, in order to reduce the possible influence of ions on the doping of semiconductors in the electrolyte solution, the researchers further modified the surface with immobilizing phospholipid (PL) molecules, where the deposited PL molecular layers were amphiphilic molecules with a non–polar nature, and the diffusion of ions through the membrane was minimized, ultimately limiting ion doping and maintaining good field–effect performance (Figure 4b) [25].

Mulla et al. used the spin–coating method to functionalize the PBTTT surface. A thin layer of polyacrylic acid (PAA) was spin–coated directly onto the PBTTT surface and then carboxyl functional group was generated by the UV–assisted cross–linking process to bind with biotinylated phospholipid (B–PL) containing membranes (Figure 4c) [26]. Sun et al. developed a novel material, 2,6–bis(4–formylphenyl)–anthracene (BFPA), to modify the poly{3,6–dithiophen–2–yl–2,5–di(2–octyldodecyl) pyrrolo [3,4–c] pyrrole–1,4–dione–alt–thienylenevinylene–2,5–yl} (PDVT–8) layer, as shown in Figure 4d, and achieved the ultrasensitive and reliable detection of AFP biomarkers in human serum with a sensitivity of up to femtomolar level. In this device, the BFPA layer played the dual roles of protection and functionalization [27]. In addition, depositing gold nanoparticles (AuNPs) on the semiconductor surface as a functional layer was also a common method. As an example, Figure 4e shows the block copolymer (BCP)–templated AuNP techniques used by Bao’s group, in which hydrogen tetrachloroaurate (HAuCl_4_) precursor was added to the poly(b4–vinylpryidine) (PS–b–P4VP) micelles and was then spin–coated on UV ozone–activated DDFTTF semiconductors; a large area of highly ordered AuNPs were deposited after the PS–b–P4VP was removed [28]. The AuNPs were subsequently functionalized to provide modular attachment points for DNA aptamer [29,30].

#### 2.2.2. Chemical Functionalization Methods

**Figure 5:**
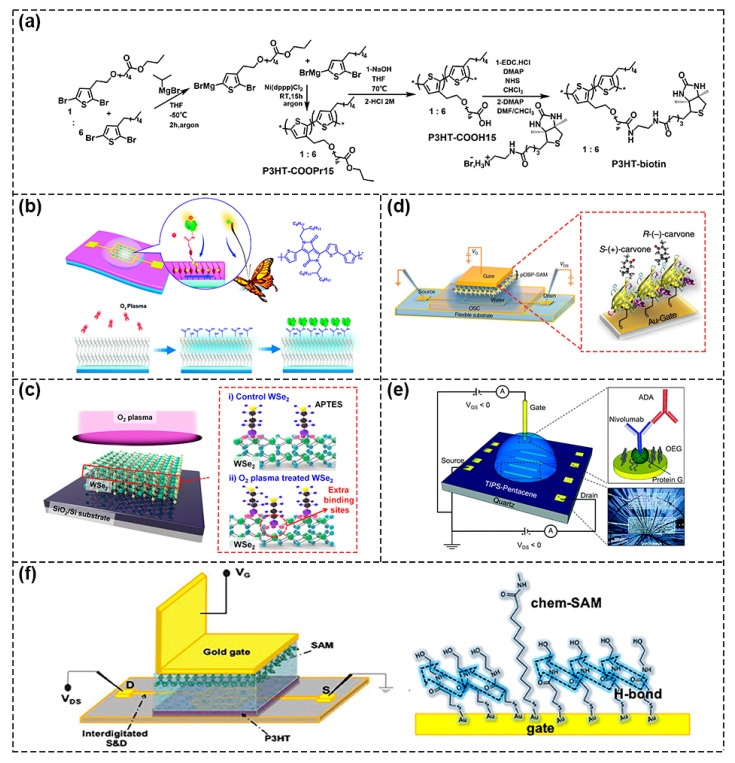
Schematic diagram of chemical functionalization methods. (**a**) The synthesis of a biotinylated polymer semiconductor consisting of carboxyl groups and biotin groups. Reproduced with permission from [31]. Copyright 2013, The Royal Society of Chemistry. (**b**) Schematic of the method of plasma–assisted–interface–grafting to introduce molecular antennas on the surface of semiconductor. Reproduced with permission from [33]. Copyright 2018, The Royal Society of Chemistry. (**c**) Schematic diagram of O_2_ plasma treated WSe_2_ flakes. Reproduced with permission from [36]. Copyright 2018 American Chemical Society. (**d**) Schematic representation of the SAMS layer on the gold surface by Au–S chemical bonds. Reproduced with permission from [37]. Copyright 2015, Macmillan Publishers Limited. (**e**) Schematic of the thiol groups of cysteine combined with Au gate to bind with Cys–protein G for detecting the F_C_ fragment of ADA. Reproduced with permission from [38]. Copyright 2021, The Royal Society of Chemistry. (**f**) Schematic diagram of the device structure using both Chem–SAM and Bio–SAM to modify the gate (**left**). Schematic of hydrogen bond network originated from Chem–SAM (**right**). Reproduced with permission from [40]. Copyright 2020, American Chemical Society.

One of the chemical functionalization methods is to introduce functional groups directly to semiconductors that have outstanding charge transport properties so that biological receptors can be immobilized onto them. Horowitz’s group synthesized a new biotinylated polymer semiconductor consisting of biotin groups to detect avidin and streptavidin (Figure 5a) [31]. However, due to the introduction of functional groups, the molecular packing was changed and weakened π–π interaction among the molecules, which affected the transport path of charges and led to a sharp deterioration in device performance [32]. There were also some researchers using techniques such as ultraviolet (UV)ozone treatment and O_2_ plasma treatment to generate a small number of defects on the semiconductor surface to serve as binding sites for biological receptors. For example, Zhu’s group used the method of plasma–assisted–interface–grafting to introduce molecular antennas on the surface of semiconductors (Figure 5b). Minimized molecular gaps and reduced boundary interactions enhanced the interaction between the semiconductor active layer and adenosine triphosphate (ATP) in solution, reaching a low detection limit of 0.1 nM [33].

The O_2_ plasma–generated oxygen–containing groups can be used to covalently tether the self–assembly membranes (SAMs), which can help to immobilize bio–sensitive probes in an efficient way [34,35]. As shown in Figure 5c, Lee et al. used O_2_ plasma to treat mechanically exfoliated tungsten diselenide (WSe_2_) flakes and then amino groups were introduced by using triaminopropyltriethoxysilane (APTES) as a silane coupling agent to immobilize bioreceptors.Compared with WSe_2_ without O_2_ plasma treatment, more surface defects were generated on the treated surface to serve as an additional binding site to hold APTES molecules. As a result of the additional binding sites of the biological receptor, sensitivity was further enhanced [36].

The other method involved using an Au gate as a sensor area, so that the SAMs layer was formed on the gold surface through the Au–S chemical bond. The bio–sensitive probes were fixed on the Au gate through the SAM layer for biological testing. Mulla et al. treated the gate region with 3–mercaptopropionic acid (3–MPA) solution to form a SAM layer, which realized the sensitive and quantitative detection of neutral enantiomers (Figure 5d) [37]. Biscarini’s research team used cysteine to functionalize the Au gate and then Cys–protein G was adsorbed through chemical bonding onto the Au surface (Figure 5e). Because G protein could combine with the F_C_ region of the antibody specifically, the biosensor had a theoretical detection limit as low as 100 fM for anti–drug antibodies (ADA) detection [38]. Macchia et al. also utilized mixed alkyl mercaptan with carboxyl groups to link onto a gold surface to form Chem–SAM and then anti–human–Immunoglobulin–G (anti–IgG) was covalently connected with carboxyl groups to form the Bio–SAM on the gate at the same time (Figure 5f). In this way, single molecule detection of IgG was realized with a millimeter–sized transistor. The suggested sensing mechanism involved a work function change, which was assumed to propagate through the network of hydrogen bonds in the gating field [39,40].

In the case of functional steps and sensing detection, the long–term stability and high reproducibility of devices are very important for obtaining accurate and reliable detection results. For example, when FET sensors are immersed in a physiological environment, the surface of the silica insulation layer may be hydrolyzed by cationic electrolytes and thus destroyed, further reducing the reproducibility of the sensor response. Therefore, surface passivation is very important to achieve high stability and reproducibility in detecting target molecules [41,42].

The interfaces of OFETs, including OSC/electrode interface, OSC/insulation interface and OSC/air interface, largely determine the performance of devices. Due to defects such as traps and grain boundaries at these interfaces, charge would be trapped, which affects the charge transport, inevitably leading to deviation from the desired behavior of devices. In addition, the loose arrangement of organic molecules also makes it easier for water and oxygen in the air to be absorbed at the OSC/air interface. Charge injection and transfer will also be affected by these active impurities adsorbed at the interface, thus affecting the final performance of the devices [43]. In view of these interface problems, different solutions have been explored to improve device performance. For example, the formation of an organic monolayer on the surface of silicon–based sensors through a Si–C bond to achieve surface passivation and chemical functionalization has been discussed in a recent review by Justin Gooding [44]. Li et al. prepared high–stability devices through interface engineering and strain balance strategy [45]. Osaka et al. developed a simple surface coating technique and successfully achieved the long–term stability of FET biosensors in water environments by coating reduced GO to the surface of a silicon dioxide insulation layer, which effectively prevented cations in the electrolyte from invading the gate insulator of FETs [46].

In addition, another aspect is seldom accounted for by researchers working on FET sensing: the sensing surface will have a point of zero charge (PZC), where no excess charge is present at the electrode surface. A recent work by Darwish [47] has perfectly demonstrated that the kinetics of surface reactions depend on the surface PZC, and the adsorption and recognition of molecules on the surface can be controlled by applying potential, which will have a significant impact on the design and operation of the FET sensing interface. Furthermore, this may become a new issue for researchers to consider when functionalizing FET sensors in the future.

### 2.3. Semiconductor Materials forActive Layers

#### 2.3.1. Two–Dimensional Materials (2D)

Since graphene was first introduced in 2004 [48], researchers have developed a wide variety of 2D materials. The thickness of 2D semiconductor materials is usually less than 5 nm and the carrier flow on the surface of the material is limited; this is conducive to achieving efficient signal acquisition and conversion because the 2D materials are directly exposed to the external environment. Because of these advantages, 2D materials have flourished in the field of FETs. Additionally, the large surface–volume ratio of the materials provides abundant modification sites for specific receptors, which is very important for FET–based biosensors [49].

**Figure 6:**
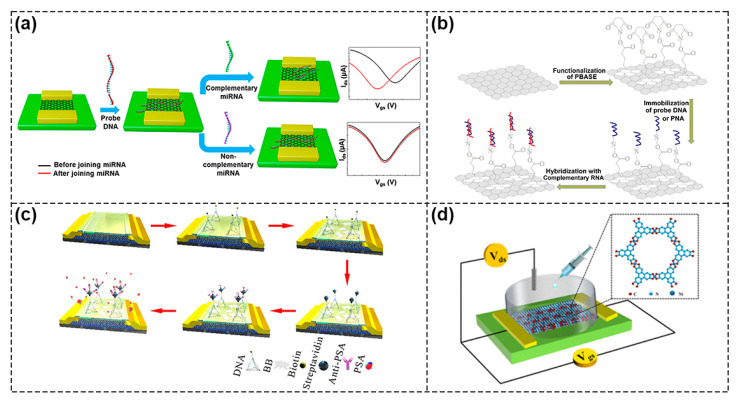
Representative FETs based on 2D materials. (**a**) Schematic of miRNA detection by GFET biosensors. Reproduced with permission from [50]. Copyright 2020, American Chemical Society. (**b**) Schematic of functionalization of PBASE as a linker on graphene surface. Reproduced with permission from [51]. Copyright 2020 Elsevier B.V. (**c**) Schematic of MoS_2_ FET with DNA functionalization. Reproduced with permission from [59]. Copyright 2021, Elsevier B.V. (**d**) Schematic of Ni–MOF–FET as biosensors for gluconic acid detection. Reproduced with permission from [65]. Copyright 2019, American Chemical Society.

- Two–dimensional layered materials;

Biosensors based on GFET have attracted much attention due to their high electron mobility, π–π stacking interactions with biomolecules and good stability. For example, Gao et al. fixed a DNA probe on the surface of the non–functionalized graphene only by using π–π interactions to achieve rapid and label–free miRNA detection within 20 min with detection limits of as low as 10 fM (Figure 6a) [50]. In order to enhance the interaction between graphene and biomolecules, some researchers have used 1–Pyrenebutanoic acid succinimidyl ester (PBASE) as a linker to treat graphene surface (Figure 6b) [51]. The pyrene group on one side of PBASE was bound to graphene through π–π interaction and the succinimide group on the other side was covalently bound to the DNA molecule. The edges and defect sites of graphene have high activity and the surface of oxidized graphene contains a large number of active epoxy groups and carboxyl groups [52], both can be used for functionalization. Therefore, Roberts et al. used 1–Ethyl–3–(3–dimethylaminopropyl) carbodiimide/N–hydroxysuccinimidesulfonate sodium salt (EDC/NHS) solution to functionalize graphene with carboxyl groups and monitored the resistance changes caused by antigen–antibody interaction in real time for the detection of Japanese encephalitis virus and avian influenza disease [53].

However, the lack of band gap in graphene results in a high leakage current of GFET biosensors, which reduces the sensors’ dynamic range. The transition metal dichalcogenide (TMD) material with X–M–X structure is composed of two atomic layers (X) and a transition metal layer (M) in between the two atomic layers (X) [54,55]. TMDs such as molybdenum disulfide (MoS_2_) and WSe_2_ exhibit a moderate band gap, which significantly reduces the leakage current in the FETs and improves detection sensitivity. Park et al. fabricated MoS_2_ FET biosensors and made rigorous theoretical simulations, and the detection limit of prostate specific antigen (PSA) was as low as 100 fg mL^−1^ with standard errors below 9% [56]. WSe_2_ FET biosensors were expected to show a good detection ability due to their high carrier mobility because high carrier mobility would affect several other performance indicators, such as current density and switching delay, in turn [57]. Hossain et al. developed a highly sensitive WSe_2_ FET biosensor for PSA detection with a very low detection limit of 10 fg ml^−1^ [58]. Due to the absorption of H_2_O and CO, the stability and detection capability of the original device would decrease and would probably lead to wrong signals. Zhang et al. used DNA tetrahedra and biotin–streptavidin (B–SA) to functionalize an MoS_2_ FETs device, which provided a more stable anchoring system for antibody–antigen (Ab–Ag) binding, so it had an ultra–high sensitivity for PSA with a detection limit of 1 fg mL^−1^ (Figure 6c) [59].

- Two–dimensional organic materials

Two–dimensional organic materials such as 2D covalent organic framework (2D COFs) and metal organic frameworks (MOF) have the advantages of periodic planar network topology, good stability, good biocompatibility, ease of functionalization and they bear abundant modification sites, which enable them to anchor a large number of specific receptors favorable to be used in biosensors [60,61,62,63,64]. For instance, Wang et al. prepared Ni –Metal–Organic Framework (MOF)–based FETs using in situ grown Ni_3_(HITP)_2_ membrane as a channel material (Figure 6d). Tightly stacked MOF films with controllable thickness were prepared by adjusting the reaction time. Due to the tightly stacked sheet structure and bare surface, the material was conducive to carrier transmission and post modification. Following this, Ni–MOF was developed as a liquid–gated device with bipolar performance and excellent response to gluconic acid in the range of 10^−6^ to 10^−3^ g mL^−1^, validating the potential of MOF–based FETs as biosensors [65].

#### 2.3.2. Polymer and Small Organic Molecule Materials

**Figure 7:**
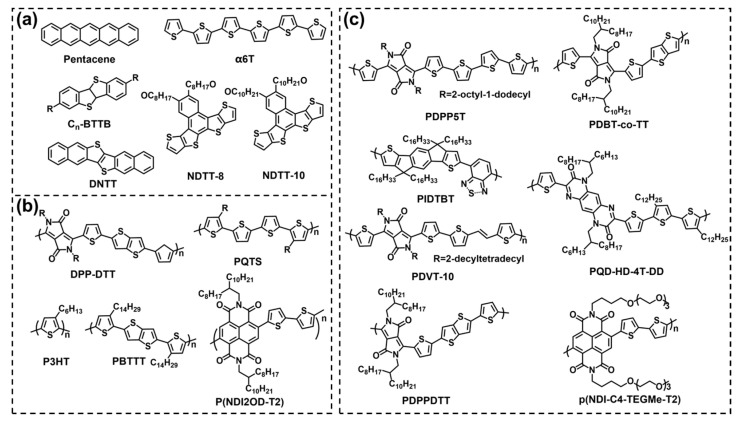
Chemical structures of organic small molecule semiconductors (**a**) and organic polymer semiconductors (**b**,**c**).

In comparison with inorganic semiconductor materials, organic semiconductor (OSC) materials have the following three advantages: (1) desired properties and functions can be obtained by simple chemical modification; (2) OSCs can be dissolved in common solvents to prepare devices by solution process methods such as spin coating and drop casting instead of the traditional vacuum deposition method, and it greatly simplifies the process of device preparation and decreases the cost; (3) there are many kinds of OSCs with good flexibility for integrating circuits and flexible displays. According to the molecular weight of the OSC materials, they can be divided into small molecule materials and polymer materials. The chemical structures of some typical OSC materials are shown in Figure 7.

**Figure 8:**
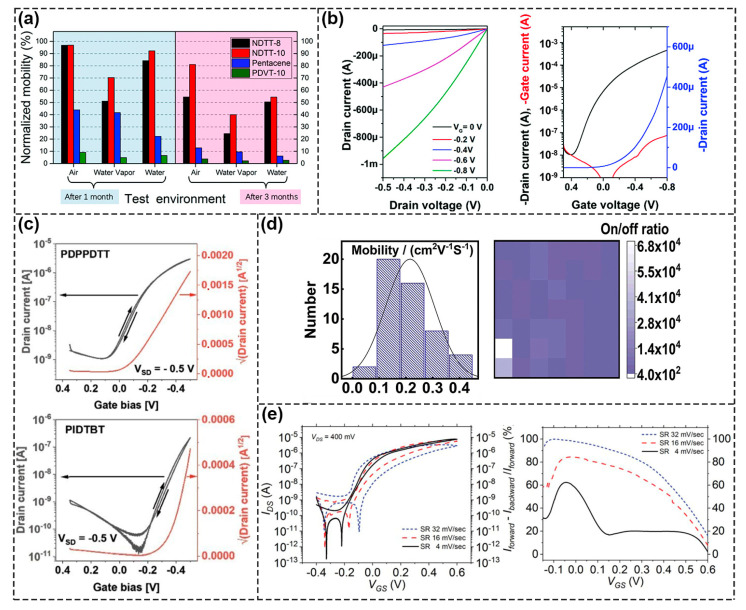
Performance of semiconductors used in FET Biosensors. (**a**) Stability testof NDTT–8 and NDTT–10 in water environment. Reproduced with permission from [67]. Copyright 2019, The Royal Society of Chemistry. (**b**) Characteristic I–V curves of PQD–HD–4T–DD polymer in water environments. Reproduced with permission from [69]. Copyright 2020, The Royal Society of Chemistry. (**c**) Representation of characteristic curves of PDPPDTT and PIDTBT transistors. Reproduced with permission from [70]. Copyright 2021, Wiley–VCH GmbH. (**d**) Saturation mobility and on/off ratio of PDBT–co–TT polymer transistors. Reproduced with permission from [71]. Copyright 2020, Elsevier B.V. I. (**e**) Characteristic I–V curves of N–type polymers, p(NDI–C4–TEGMe–T2). Reproduced with permission from [72]. Copyright 2022, Wiley–VCH GmbH.

Typical small molecule materials include pentacene, α–sexithiophene (α6T), 2,7–dialkyl[1]benzothieno[3,2–b][1] benzothiophene (C_n_BTBT), dinaphtho [2,3–b:20,30–f]thieno[3,2–b]thiophene (DNTT), and so on. For example, Song et al. prepared an extended gate OFET with pentacene as a semiconductor layer for the detection of glial fibrinous acidic protein [66]. Li et al. synthesized naphthodithieno [3, 2–b] –thiophene derivatives NDTT–8 and NDTT–10. They showed excellent water stability compared to pentacene and poly{3,6–dithiophen–2–yl–2,5–di(2–decyltetradecyl)–pyrrolo[3,4–c] pyr–role–1,4–dione–alt–thienylenevinylene–2,5–yl} (PDVT–10) polymers. After 90 days in water, the μ of the carrier remained above 50% (Figure 8a) [67]. However, because the performance of most small molecule OFETs degraded rapidly once they were exposed to moisture, they were not suitable for the detection of biomolecules in liquid environments [68]. In order to further improve the stability of devices, polymer semiconductor materials were applied. Typical polymer materials include poly[2,5–(2–octyldodecyl)–3,6–diketopyrrolopyrrole–alt–5,5–(2,5–di(thien–2–yl)thieno[3,2–b]thiophene)](DPP–DTT), poly(3–hexylthiophene) (P3HT), poly[2,5–bis(3–tetradecylthiophen–2–yl)thieno[3,2–b]thiophene](PBTTT), poly[[1,2,3,6,7,8–hexahydro–2,7–bis(2–octyldodecyl)–1,3,6,8–dioxobenzo[lmn][3,8]phenanthroline–4,9–diyl][2,2′–bithiophene]–5,5′–diyl] [P(NDI2OD–T2)], diketopyrrolopyrrole–based π–conjugatedcopolymer (PDPP5T) and so on. As shown in Figure 8b, Leong’s group fabricated high–performance WGOFETs using PQD–HD–4T–DD polymer and the average μ was 9.76×10^−3^ cm^2^ V^−1^ s^−1^, I_on_/I_off_ was 4.41×10^4^ [69]. Doumbia et al. synthesized two D–A polymers, (poly[2,5–(2–Octyldodecyl)–3, 6–Diketopyrrolopyrrole–alt–5,5–(2,5–di(thien–2–yl) thieno) [3,2–b] thiophene)] (PDPPDTT) and indacenodithiophene–co–benzothiadiazole (PIDTBT) for WGOFETs. The I_on_/I_off_ were 3 × 10^3^ (PDPPDTT) and 2 × 10^4^ (PIDTBT), respectively. The μ of PDPPDTT was 0.18 cm^2^ V^−1^ s^−1^ and PIDTBT was 0.16 cm^2^ V^−1^ s^−1^ (Figure 8c) [70]. Sun et al. synthesized π–conjugated polymer material PDBT–co–TT for WGOFETs with an average mobility of 0.22 cm^2^ V^−1^ s^−1^ and a switching ratio of 5.13 × 10^3^ (Figure 8d), which exceeded most of those reported WGOFETs to date [71]. Compared to P–type polymers, N–type polymers were affected heavily by air/water and had low performance, so they were not widely used in biosensors. Caironi et al. presented the first example of an N–type electrolyte–gated organic transistor based on an inkjet printing polymer, p(NDI–C4–TEGMe–T2) (Figure 8e).The device showed excellent working stability of more than 18 h and a switching ratio of more than 10^4^ [72]. In terms of the material, they should have a suitable energy level and a good match with the work function of the source and drain to facilitate the effective injection and output of charge carriers, resulting in different detection performance (Table 1).

## 3. Application

### 3.1. Immunoassay Based on Single Biosensor

At present, serology and viral nucleic acid testing are two main diagnostic methods for COVID–19 [73,74,75], but they cannot meet the requirements of diagnostic accuracy and detection speed at the same time. It is becoming more and more important to develop biosensing devices with high sensitivity, fast detection speed and less volume, which is where researchers have concentrated a lot of effort.

**Figure 9:**
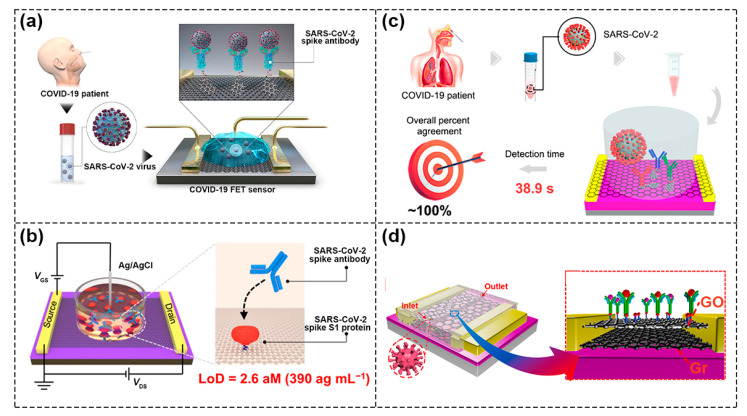
COVID–19 detection based on different FET biosensors. (**a**) Schematic of the SARS–CoV–2 spike antibody coupled to graphene sheet for detecting SARS–CoV–2 virus. Reproduced with permission from [76]. Copyright 2020, American Chemical Society. (**b**) Schematic of a GFET biosensor modified with spike S1 protein for detecting SARS–CoV–2 spike antibody. Reproduced with permission from [1]. Copyright 2021, American Chemical Society. (**c**) Schematic of the multi–antibodies FET sensors for detecting SARS–CoV–2. Reproduced with permission from [78]. Copyright 2021, American Chemical Society. (**d**) Schematic of GO/Gr heterostructure biosensors for SARS–CoV–2 detection. Reproduced with permission from [79]. Copyright 2021, Elsevier B.V.

Seo et al. reported on a FET biosensor for detecting SARS–CoV–2 virus in clinical samples, in which the SARS–CoV–2 spike antibody was coupled with a graphene sheet and used as sensing area (Figure 9a). It was able to detect SARS–CoV–2 spike protein in the clinical transport medium of 100 fg/mL [76]. Wei’s group also developed a GFET biosensor modified with spike S1 protein (Figure 9b). Through the specific binding of SARS–CoV–2 antibody and S1, the conductance in graphene channels changed, and the ultra–low detection limit of SARS–CoV–2 antibody reached 2.6 aM [1]. The research group also tried to use DNA probes as recognition elements; however, conventional flexible SS DNA probes would aggregate and entangle at the sensing interface of conductive channels, leading to the inactivation of SS DNA probes, thus researchers used GFET and Y–shaped DNA dual probes (Y–dual probes) to detect SARS–CoV–2 nucleic acid. Due to the synergistic effect of probe sites targeting the ORF1ab and N gene regions, the biosensor had a high recognition rate for SARS–CoV–2 nucleic acid and reached a detection limit of three copies in 100 μL solution [77]. At present, most research on biological target analytes is focused on proteins including antigens, enzymes, etc., which are generally detected directly without an amplification process, leading to less accuracy than polymerase chain reaction (PCR). As shown in Figure 9c, Wei et al. demonstrated a multi–antibody FET sensor and successfully detected SARS–CoV–2 in artificial saliva with a detection limit of 3.5 × 10^−17^ g/mL and a detection limit of 0.173copies μL^−1^ in nasopharyngeal swabs [78]. In Figure 9d, Gao et al. fabricated biosensors using a van der Waals heterostructure of graphene and graphene oxide (GO) [79]. Compared with the GFET biosensor, the sensitivity for SARS–CoV–2 protein detection of the biosensors with GO/Gr heterostructure was increased threefold. This was mainly due to the fact that GO formed a uniform protective layer, which could prevent external ions from directly contacting the surface of graphene. At the same time, due to the formation of heterojunctions, the efficiency of electron exchange was improved through interface coupling and the charge mobility of the device was further improved. The advantage of 2D–layered materials is that they can be further integrated with other materials to form a special heterojunction at the atomic scale, which opens up new opportunities for constructing new biosensor components.

### 3.2. Integrated into Array for Multiplexing

**Figure 10:**
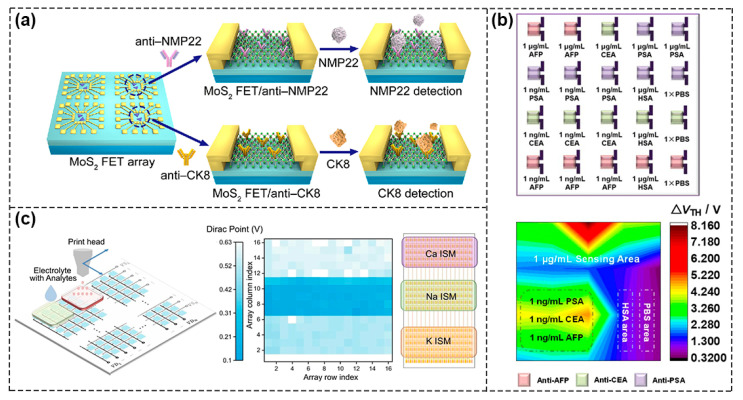
Integration of FET biosensors into array for multiplexing. (**a**) Schematic of FET sensor arrays based on MoS_2_ nanosheets for simultaneous detection of multiple bladder cancer biomarkers. Reproduced with permission from [81]. Copyright 2020, Science China Press and Springer–Verlag GmbH Germany, part of Springer Nature.(**b**) Simultaneous determination of three biomarkers using a FET sensor array. Reproduced with permission from [82]. Copyright 2022, American Chemical Society. (**c**) Diagram of 16 × 16 sensor unit (**left**). Color map of Dirac points for three kinds of ion–sensing unit (**right**). Reproduced with permission from [83]. Copyright 2022, the author(s).

The variability of devices due to uneven features during the process of material synthesis and device fabrication techniques is a critical concern in detecting single analytes, which may lead to certain errors. Li et al. constructed 120 silicon nanowires (SiNW) channels as the sensing area for sensitive detection of PIK3CA E542K ctDNA in parallel and the prepared SiNW FET sensors had good specificity and repeatability with an ultra–low detection limit of 10 aM [80]. The composition of real clinical samples is very complex and detecting a single analyte is far from meeting the need for early diagnosis of specific diseases. Therefore, the development of an efficient approach to simultaneously detect multiple markers and realize high–throughput screening is extremely necessary. With the rapid development of device miniaturization and integration, FET sensor arrays with multi–channel sensing units can be constructed to detect a variety of biomarkers so as to improve detection sensitivity and accuracy and to promote clinical application. As shown in Figure 10a, Yang et al. fabricated a FET biosensor composed of four sensing windows based on MoS_2_ nanosheets, in which each module can be used to detect a single biomarker without interfering with the other. At the same time, each sensing window contained multiple parallel sensing units so as to achieve multi–channel detection. Bladder cancer biomarkers, nuclear matrix protein 22 (NMP22) and cytokeratin 8 (CK8), were detected simultaneously with detection limits of 0.027 and 0.019 aM, respectively, suggesting that properly designed multi–channel sensor arrays can be routinely used for detection with high sensitivity and accuracy [81]. Sun et al. integrated the prepared DMP [5]–COOH molecules as signal amplifiers with OFET devices and the sensing array was divided into different detection areas, which realized synchronous and immediate detection of three tumor markers with ultra–high sensitivity at aM level (Figure 10b) [82].Furthermore, as shown in Figure 10c, a graphene–based sensor array platform that consisted of more than 200 (16 × 16) integrated sensing units was constructed by Xue et al. The sensor chip was designed as three separate regions to enable the detection of potassium, sodium and calcium ions in complex solutions, such as artificial urine and artificial eccrine perspiration. The way to functionalize the graphene surface was by depositing three different ion–selective membranes (ISMs) using a 3D printing machine. Then, they further utilized the stochastic Forest algorithm model to demonstrate ion type classification, concentration prediction and disease diagnosis, thereby enhancing the reliability of the data. This also demonstrated the importance and effectiveness of combining experimental testing with machine model learning [83].In addition, the FET sensors could also be used in biomimetic human sensory systems. Kwon et al. reported on an artificial multiplex super bioelectronic nose (MSB–nose) using highly homogeneous graphene micropatterns (GMs) with two different human olfactory receptors attached to GMs as bio–probes [84]. It mimicked the human olfactory sensory system and had high performance in odor discrimination from mixtures. In addition, Ahn et al. developed GFET–based dual biological electronic tongues (DBTs) for the simultaneous detection of umami and sweet tastes, thus opening up new ways of mimicking human complex biomimetic systems and demonstrating the great potential of FET–based biosensors [85].

### 3.3. Integrated with Microfluidicsfor LAB–on–CHIP

Lab-on-chip (LOC) is a kind of device that integrates laboratory functions on a chip whose size is from a square millimeter to a few square centimeters. LOC has facilitated the development of handheld, miniaturized medical diagnostic test platforms. Integrating FET biosensors with microfluidic devices is an attractive direction in LOC [86].

**Figure 11:**
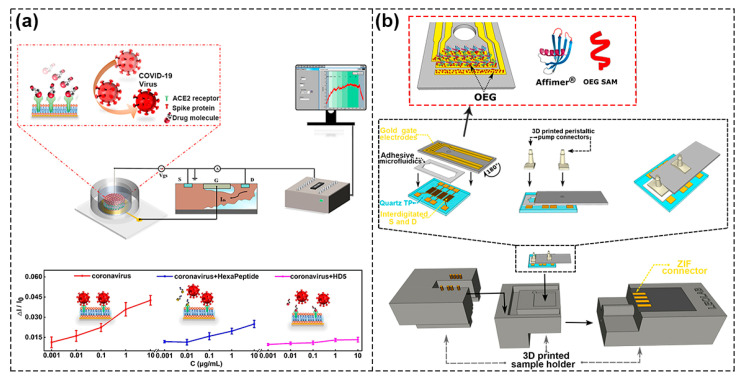
Schematic of integrated FET biosensors with microfluidic for lab–on–chip. (**a**) Schematic of biosensor chip modified by SLB (**top**) and the inhibitory response of two different drugs to the interaction between coronavirus and ACE2 receptor (**bottom**). Reproduced with permission from [89]. Copyright 2022, American Chemical Society. (**b**) Schematic of a lab–on–chip multi–gates organic transistor based on 3Dprinting and modified multi–gates in the red dotted box. Reproduced with permission from [91]. Copyright 2020, American Chemical Society.

Dai et al. realized the simultaneous detection of penicillin G and urea by designing urease–encoded and penicillinase–encoded polyethylene glycol hydrogels. The hydrogels were used as the biometric identification module to directly contact the graphene channel, in which they can be freely assembled and disassembled, which made the programmable sensing function of FET sensor chip systems possible [87]. Kim et al. combined the antibiotics conjugated graphene micropattern FET (ABX–GMFETs) with a microfluidic chip to detect dual bacterial Gram–positive bacteria (GPB) and Gram–negative bacteria (GNB) [88]. As shown in Figure 11a, Zhou et al. prepared an extended–gate FET biosensor chip modified with a supported lipid bilayer (SLB) and angiotensin–converting enzyme II (ACE2) receptor, where SARS–CoV–2 binding with ACE2 receptors infected host cells and SLB was used to provide the cell–simulated environment. The aim was to study the interaction between SARS–CoV–2 and cell membrane so as to facilitate the screening of effective anti–coronavirus drugs. The detection results showed that the presence of two different drugs had an effect on the interaction between coronavirus and the ACE2 receptor, with weak inhibition by hexapeptide and strong inhibition by HD5 peptide. The integrated system could translate the interaction between biological target analytes and receptors into real–time charge signal, so as to realize effective screening of therapeutic drugs [89]. Hajian et al. prepared CRISPR–Chip by modifying graphene surface with CRISPR–Cas9 complex. The chip could conveniently, rapidly, and selectively detect target sequences of CRISPR–Cas9′s gene and had the potential to extend the boundaries of digital genomics [90].

In addition to the rapid detection of biomolecules, LOC can take advantage of a smaller sample volume and can conduct several sample tests simultaneously to assess the occurrence of non–specific interactions and minimize the chance of false positives. As shown in Figure 11b, Parkula et al. integrated multi–gates EGOFETs and a single reservoir microfluidic system in a 3D–printed sample box and detected binding events occurring at the gate–electrolyte interface in a 6.5 μL microfluidic channel with pM accuracy. To be specific, the proinflammatory cytokine tumor alpha (TNFα) samples were detected by three gates simultaneously, and the fourth electrode was used as a reference electrode to assess whether the detection response had to be attributed to the sensing event itself, which reduced the influence of non–specific adsorption [91]. It was a major step forward in the robustness and cost–effectiveness of detection, as it was able to increase the statistics of biomarker detection in the smallest sample volume and meet the trend of personalized medicine, which are guaranteed in biosensor applications at point of care (PoC).

### 3.4. Integrated with other Sensors for Multifunctional Applications

**Figure 12:**
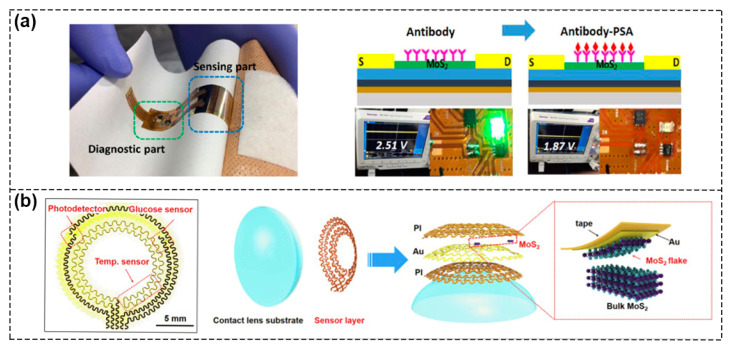
Schematic of multifunctional sensing systems. (**a**) The devices with system–level integration of flexible MoS_2_ FET biosensors, read–out circuits and LEDs. Photograph of an epidermal skin–type MoS_2_ biosensor system (**left**). Optical images of the LED indicator biochip for PSA detection (**right**). Reproduced with permission from [92]. Copyright 2017, Tsinghua University Press and Springer–Verlag Berlin Heidelberg. (**b**) Optical image of the serpentine mesh sensor system, including a photodetector, a temperature sensor and a glucose sensor, and schematic illustration of the different layers of smart contact lens structure attached to an eyeball. Reproduced with permission from [93]. Copyright 2021, Elsevier Inc.

The integration of different sensors on the same chip allows multiple functions to be performed in a small volume. High integration means more functionalities in a smaller size with a lighter weight, which can meet the requirements of the next generation smart system. As shown in Figure 12a, Yoo et al. reported a flexible biochip within which a MoS_2_ FET biosensor, readout circuit, and light–emitting diode (LED) were integrated. When 1 μg·mL^−1^ PSA was fixed on the MoS_2_ surface, the corresponding off current increased and the output voltage amplified, which led to the lighting up of the LED indicator. Following this, when 100 pg·mL^−1^ PSA was bound to the immobilized antibody, the off current decreased, the output voltage dropped to 1.87 V, and the LED turned off, realizing the real–time and POC diagnosis of prostate cancer markers [92]. Stretchable and bendable devices integrated with multifunctional biosensors or devices implanted in the human body are able to sense physiological signals and environmental conditions in real time without affecting normal body movement. Guo et al. demonstrated a multifunctional smart contact lens sensor system based on ultrathin MoS_2_ transistors including a photodetector to receive optical information, a glucose sensor to directly monitor glucose levels in tears, and a temperature sensor to diagnose underlying corneal diseases (Figure 12b) [93].

## 4. Summary and Prospect

FET biosensors have made exciting progress in terms of device structure, material synthesis, device manufacturing, microfluidic industry–compatible technologies and multifunctional integrated applications. FET devices can detect a large variety of biomolecules/entities, from proteins to viruses, to bacteria, and cells in the body even at very low concentrations, thus opening up possible applications for almost any pathology and showing fresh vitality in wearable electronic devices and other fields [94,95].

Despite the fact that FET–based biosensors have the advantages of high sensitivity and fast detection speed, there are some aspects that still need to be improved and developed in the FET–based biosensors system. (1) Biomolecular immobilization technology: On the one hand, suitable methods to achieve stable and reliable immobilization of biomolecules on the sensor surface are still in high demand. On the other hand, methods to improve the density, the uniformity and orderly arrangement of the immobilized biomolecules on the sensing surface need to be developed to improve the sensing performance. (2) Selectivity and sensitivity: In addition to the target biomolecules, some non–target analytes also could be attached to the biosensor interface and will generate interference signals to the biosensors. Therefore, it is essential to develop methods to prevent the attachment of non–specific adsorbates, such as passivation of the excess functional groups by proper reagents. Designing masks and optimizing channel size can also play a role in improving sensitivity. (3) Reusability: Currently, most sensors are single use only, but the preparation of biosensors with a regenerative ability has a wider prospect in real–time applications. For example, Zhao et al. used Nafion solution to prepare a reproducible FET biosensor and realized the reusability of a single device [96]. (4) Microfluidic techniques for POC diagnosis have been shown to be effective in reducing sample size, testing cost, and time. Current leakage and power consumption problems must be considered in preparing microarrays, and integrating FET biosensors with microfluidic devices requires proper design of the FET structures, such as selecting dielectric layers with high k values to detect analytesat a low operating voltage(<1 V), etc. (5) Existing FET biosensors are mainly focused on in vitro detection of biological species, whereas bioelectronic devices are developing towards implantable, wearable and non–invasive measurement. Therefore, it is imperative to develop excellent biocompatible and flexible FET biosensors.

In addition, the transformation of this emerging technology from the laboratory to commercial production still requires the joint efforts of researchers and industrial circles. Developing and constructing FET biosensors of a small size, with low cost and commercial availability still presents great challenges, including: (1) Cost factor: researchers need to consider inexpensive methods and materials for mass production of standardized sensors. (2) Poor reliability: in addition to the cost factor, poor reliability is also a factor that cannot be ignored. In the process of commercialization, the inevitable quality problems in the large–scale manufacturing of devices must be taken into account. (3) Real–time communication capability: realizing real–time and remote data collection and processing for each individual through the Internet and to realize health monitoring and environmental testing, the balance of sensor performance and other parameters must be taken into account [97,98]. Furthermore, FET–based biosensors serve as an outstanding tool to bridge the worlds of electronics and biology, and further development of new sensing applications remains to be explored.

## Figures and Tables

**Table 1:** Semiconductor materials used for FET biosensors.

Characteristic	Semiconductor	Mobility (cm^2^V^−1^s^−1^)	I_on/off_	Analyte	Detection Limit	Times (min)	Sensitivity	Ref
① High surface–volume ratio, high theoretical carrier velocity (~10^6^ m/s) and mobility; ② Zero band gap, large leakage current, reducing the dynamic range of the sensor, sensitive to external conditions, such as electric field and foreign doping impurities.	Graphene			SARS–CoV–2 antibody	10^−18^ M	<2	4%	1
				DNA	1 nM	minutes	N/A	15
				miRNA	10^−15^ M	20	5.99 mV/decade	50
				RNA	0.1 aM	minutes	14.8	51
		3.79 (hole)	3100	Hg^2+^	16 pM/L	minutes	N/A	52
		3.78 (electron)		JEV/AIV	1 fM/10 fM	minutes	N/A	53
			N/A	SARS–CoV–2 antigen	2.42 × 10^2^ copies/mL	>1	N/A	76
		>10,000 * (Room temperature)		SARS–CoV–2 Nucleic acid	0.03 copy/μL	~1	N/A	77
				SARS–CoV–2 protein	~8 fg/mL	minutes	12.8mV/decade	79
				K^+^ Na^+^ Ca^+^	~100 μM	N/A	−54.7 ± 2.90 −56.8 ± 5.87 −30.1 ± 1.90 mV/decade	83
				DNA	1 nM	N/A	30.1mV/decade	86
				Nucleic acid	1.7 fM	~2.5	N/A	90
				IFN–γ	880 fM	minutes	N/A	97
① Adjustable intrinsic band gap, high carrier mobility, large switching ratio, low leakage current; ②Sensitive toexternal conditions;	WSe_2_	133	~10^5^	PSA	10 fg/mL	minutes	2.6	58
	WSe_2_	N/A	N/A	Glucose	10 mM	N/A	2.87 × 10^5^ A/A	36
	MoS_2_	N/A	~10^6^	PSA	100 fg/mL	minutes	N/A	56
	MoS_2_	N/A	N/A	PSA	1 fg/mL	~4	0.05%	59
	MoS_2_	19.4	~10^2^	NMP22/CK8	0.027/0.019 aM	N/A	N/A	81
	MoS_2_	83.5	~10^6^	PSA	1 pg/mL	2~3	N/A	92
	MoS_2_	9.18	~10^7^	Glucose	N/A	N/A	N/A	93
① Easy modification, adjustable energy band, high flexibility, easy solution processing, good hydrophobicity; ② Polymerization generally takes place at high temperature and consumes energy, low carrier mobility.	P3HT–COOH	0.5 ± 0.12	~10^3^	DNA	N/A	minutes	N/A	14
	DDFTTF	~0.35	2 × 0^3^	DNA	N/A	minutes	N/A	22
	P3HT	0.006	N/A	D–Phe	10^−13^ mol/L	N/A	N/A	18
	P3HT	10^−3^	204 ± 91	SA	10 nM	45s	N/A	25
	PBTTT	N/A	N/A	α–casein	0.22 ppm	N/A	N/A	17
	PBTTT	N/A	N/A	BSA	6 × 10^−13^ M	<15	N/A	35
	PBTTT	~0.02	10^2^−10^3^	SA	10^−11^ M	minutes	N/A	26
	PBTTT–C14	(1.1 ± 0.2) × 10^−1^	N/A	pOBP protein	50 pM	N/A	N/A	37
	PDVT–8	0.18	~10^5^	AFP	4.5 fM	40	2.7%	27
	DDFTTF	0.25	2 × 10^3^	Hg^2+^	100 μM	N/A	N/A	29
	P3HT–biotin	~10^−4^	~80	Streptavidin	N/A	minutes	2%	31
	PDPP3T	0.3~0.6	~10^3^	ATP	0.1 nM	minutes	N/A	33
	PDBT–co–TT	0.22	5.13 × 10^3^	AFP	0.15 ng/mL	45	N/A	71
	PDBT–co–TT	2.07	~10^6^	AFP/ CEA	0.176 pM/ 65 fM	minutes	N/A	21
	PDBT–co–TT	~0.1	~10^3^	AFP/CEA/ PSA	4.75 aM	N/A	N/A	82
① Clear structure, easy to purify, ② Poor film formation, not conducive to large area preparation. Sensitive to external conditions;	α 6T	4 × 10^−2^	10^2^−10^3^	Penicillin	5 μM	minutes	50 μV/μM	34
	Pentacene	0.116	~10^6^	BSA	N/A	N/A	N/A	23
	Pentacene	0.69 ± 0.07	26.0 ± 5.7	GFPA	1.0 ng/mL	minutes	N/A	66
	Pentacene	N/A	N/A	TNF **α**	3 pM	N/A	N/A	91
	TIPS–pentacene	N/A	N/A	ADAs	10^−13^ M	minutes	10^11^ M^−1^	38

Notes: ① represents advantages and ② represents disadvantages of different materials. The asterisk (*) represents the theoretical value.

## Data Availability

Not applicable.
